# Association between Dietary Patterns and Cardiovascular Risk Factors among Middle-Aged and Elderly Adults in Taiwan: A Population-Based Study from 2003 to 2012

**DOI:** 10.1371/journal.pone.0157745

**Published:** 2016-07-01

**Authors:** Miriam Adoyo Muga, Patrick Opiyo Owili, Chien-Yeh Hsu, Hsiao-Hsien Rau, Jane C-J Chao

**Affiliations:** 1 School of Nutrition and Health Sciences, College of Public Health and Nutrition, Taipei Medical University, Taipei, Taiwan; 2 International Health Program, Institute of Public Health, National Yang-Ming University, Taipei, Taiwan; 3 Department of Information Management, National Taipei University of Nursing and Health Sciences, Taipei, Taiwan; 4 Master Program in Global Health and Development, College of Public Health and Nutrition, Taipei Medical University, Taipei, Taiwan; 5 Graduate Institute of Biomedical Informatics, College of Medical Technology, Taipei Medical University, Taipei, Taiwan; 6 Nutrition Research Center, Taipei Medical University Hospital, Taipei, Taiwan; Weill Cornell Medical College in Qatar, QATAR

## Abstract

**Background:**

Cardiovascular disease (CVD) is one of the leading causes of mortality and loss of disability-adjusted life years in developed countries. This study derived a dietary pattern using an *a priori* method and additionally derived dietary patterns using *a posteriori* methods, and assessed the relationship with CVD risk factors in Taiwanese middle-aged and elderly adults.

**Methods:**

Cross-sectional analyses of 62,965 subjects aged 40 years and above from the Mei Jau (MJ) database collected between 2003 and 2012 in Taiwan. Diet was assessed using a 22 item semi-quantitative food frequency questionnaire. Using this information, three dietary patterns were generated. The *a priori* diet was labeled the Taiwanese dietary pattern and was derived using hypothesized effect of 22 food groups, while two *a posteriori* dietary patterns, “vegi-fruits” and “meat-processed”, were derived using principal component analysis. The association between dietary patterns and a range of CVD risk factors (i.e. blood lipids, blood glucose and C-reactive protein) was evaluated using linear regression.

**Results:**

The results showed that high intake (Q5, quintile 5) of Taiwanese diet was negatively associated with CVD risk factors at (*p* < 0.001, model 3), but not with triacylglycerol. In addition, high intake of vegi-fruit dietary pattern (Q5) was negatively associated with CVD risk factors (*p* < 0.001), but not with high-density lipoprotein, while high consumption of meat-processed dietary pattern (Q5) was positively associated with CVD risk factors (*p* < 0.001), but negatively related with triacylglycerol in Q3 level and no association with C-reactive protein.

**Conclusion:**

A negative association was observed between Taiwanese or vegi-fruit dietary patterns and CVD risk factors, while a positive association was found between meat-processed dietary pattern and CVD risk factors. The findings suggested that a diet rich in vegetables and fruits has a beneficial effect in the management of CVD risk factors.

## Introduction

Cardiovascular disease (CVD), one of the leading causes of death, claims more lives than all types of cancers combined [1]. Furthermore, CVD has been shown to result in premature death and loss of disability-adjusted life years [1–3]. However, diet is considered to be one of the most efficient and effective ways in the management of CVD [3]. The evidences have shown a significant relationship between food patterns and chronic diseases. A diet high in vegetable and fruits was associated with lower risk of non-communicable diseases (NCDs), whereas a diet high in meat was associated with higher risk of NCDs [4–7]. Therefore, overall assessment of dietary patterns with combinations of food groups in nutritional epidemiology is a better complementary or alternative method for predicting the relationship between dietary intake and the risk of chronic diseases than using single food consumption [8]. Two methods are commonly used to derive dietary patterns–*a priori* and *a posteriori*. *A priori* method uses dietary indices to derive dietary patterns as a composite score of foods, nutrients or both. *A posteriori* method uses statistical techniques such as principal component analysis (PCA), factor analysis or cluster analysis to derive dietary patterns. *A priori* method is theoretical while *a posteriori* method is empirical and uses multivariate techniques [9]. *A priori* method reflects the health effect of a broader definition of food items using pre-established food groups based on the most common food groups (i.e. dairy, fruits, grains, meats, confections, vegetables, etc.) or from presumption, while *a posteriori* method such as PCA provides a linear combination (components/patterns) of the food groups based on the data provided on food consumption of the study population [8, 10]. Previous dietary pattern studies conducted in different populations, using either *a priori* or PCA or both to assess diets and CVD risk factors, have found that the diets high in fruits and vegetables or fiber were negatively associated with CVD risk factors; while diets high in meat and processed foods were positively associated with CVD risk factors [10–12].

However, the need for further research on global health effect of dietary patterns among middle-aged and older adults has been supported [13]. Since there are a lot of differences in nutrient intake as a result of eating various food items, it is not appropriate to focus only on one particular nutrient or dietary component in the determination of health effect among middle-aged and elderly adults [14–16]. Therefore, this study considered the complexity in dietary habits and its related health effect, and the results can be applied to design and implement health policy for public health promotion. Nevertheless, there is still limited literature on the dietary patterns of a free-eating population in Taiwan and how they relate to major CVD risk factors; and hence, the aim of this study was to identify the dietary patterns of middle-aged and elderly Taiwanese and to assess the relationship with CVD risk factors such as blood lipids, C-reactive protein and blood glucose.

## Materials and Methods

### Study population and sources of data

**Fig 1:**
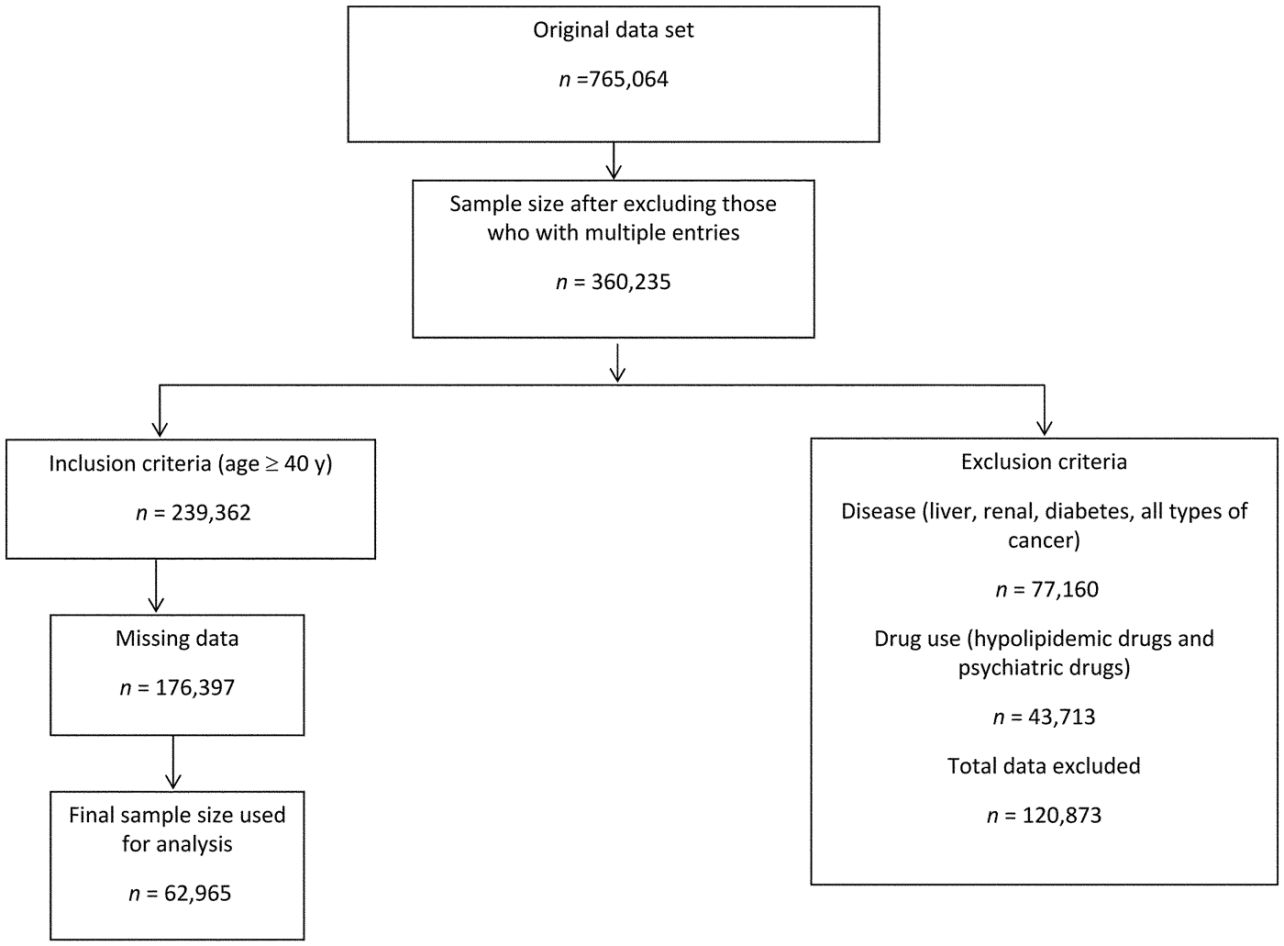
Sampling flow chart and sample size. The sampling flow chart for the association between dietary patterns and cardiovascular disease risk factors among the adults aged ≥ 40 years in Taiwan.

This cross-sectional study was conducted with a sample collected by the Mei Jau (MJ) Group, an independent health screening and management institution, in Taipei, Taiwan. The MJ Group has eight health screening centers in Asia, of which four of its centers are found in Taiwan (Taipei, Taoyuan, Taichung, and Kaohsiung). All the subjects signed the consent form to agree the data without personally identifiable information used for the purpose of academic research only before undergoing health screening. The study design was approved in its entirety by Taipei Medical University-Joint Institutional Review Board. Upon visiting the health screening center a structured questionnaire was issued to the participants to collect information on the demographics, lifestyle, medical history, diet, and exercise. The information retrieved from the database included 765,064 adults aged 40 years and above who visited the MJ health management institution center for health screening between 2003 and 2012. The data were screened for multiple entries of participants who visited the center more than once, and hence 404,829 entries were excluded. From the remaining participant (*n* = 360,235), those with chronic diseases (*n* = 77,160) such as cancer, liver disorders, renal disease and diabetes mellitus were excluded. Furthermore, participants with psychiatric illness and those using any form of lipid-lowering drugs (*n* = 43,713) were also excluded because the medication would confound the results of the study leaving 239,362 participants for the analyses (Fig 1). These health conditions were generally required dietary modification, and their dietary behavior could not reflect a free-eating population in Taiwan. The demographic characteristics such as sex, age, education (< high school, high school, and > high school) and marital status (never married, married, widow/divorced) and the information on lifestyle factors such as smoking (yes/no), drinking (yes/no), and physical activity (yes/no) were also obtained using the self-reported questionnaire during health screening.

### Cardiovascular risk factors

The outcome measures of CVD risk factors included blood lipids such as triacylglycerol (TG), total cholesterol (TC), high-density lipoprotein (HDL-C) and low-density lipoprotein (LDL-C) as well as fasting glucose and C-reactive protein (CRP). Before blood was drawn for laboratory tests, the participants were required to fast overnight (12–14 hours). The blood specimens were then collected after a questionnaire on the demographics, lifestyle, medical history, diet, and exercise was completed. The blood lipids (TG, TC and HDL-C) and fasting glucose levels were directly measured using the reagents from Randox Laboratories Limited, while LDL-C level was calculated using the Friedewald formula (LDL-C = TC- [HDL-C + TG/5] mg/dl) [17]. The CRP was directly measured using a reagent from Fortress diagnostics. The blood test was repeated to ensure that the measurement was accurate. All the blood samples were analyzed at the MJ Health Management Institution’s central laboratory. Clinically, the normal levels of blood lipids are TG less than 150 mg/dL (1.695 mmol/L), TC less than 200 mg/dL (5.18 mmol/L), LDL-C less than 100 mg/dl (2.59 mmol/L), and HDL-C greater than 40 mg/dL (1.036 mmol/L), while CRP and fasting glucose are considered normal if less than 1.0 mg/L (9.52 nmol/L) and 100 mg/dL (5.556 mmol/L), respectively [18, 19]. However, since we examined a linear relationship, these cut-off-points were not used in our analyses. Other CVD risk factors including current CVD status (yes/no), body mass index (BMI, kg/m^2^), waist circumference, systolic and diastolic blood pressure were also investigated. The body weight of the participants was measured using electronic scales, while the height measurements were obtained using aluminum anthropometers.

### Dietary assessment

Diet was assessed using a standardized and validated semi-quantitative food frequency questionnaire (FFQ) that was developed by the MJ Global Health Management Service [20, 21]. The initial questionnaire had 85 closed-ended questions on individual food items consumed by the participant at different meal times in the month prior to the data collection; which were then categorized into 22 non-overlapping food groups based on a hypothesized health effect and similarity as defined in other studies [10, 11, 22]. The frequency of food consumption and servings was assessed in terms of consumption and servings per day or per week. In the questionnaire, light or dark colored vegetables, root vegetables, and fried vegetables or vegetables with salad dressing had five response options such as < 0.5 bowl/day, 0.5–1 bowl/day, 1–1.5 bowl/day, 1.5–2 bowls/day, and 2 bowls/day (a bowl = 11 cm in diameter). Fruits and rice and noodle products had responses such as < 1 serving/day, 1–2 servings/day, 2–3 servings/day, 3–4 servings/day, and ≥ 4 servings/day; while intake of other food items were responded to as < 1 serving/week, 1–3 servings/week, 4–6 servings/week, 1 serving/day, and ≥ 2 servings/day. Each question described the definition of 1 serving of food and gave examples of food, such as how many servings of eggs do you eat? (1 serving is equivalent to 1 chicken or duck egg or 5 quail eggs).

### *A priori* (Taiwanese) dietary pattern

The *a priori* dietary pattern (labeled the “Taiwanese”) was created on a hypothesized effect on CVD risk factors with food groups categorized as beneficial (n = 8), adverse (n = 11), and neutral (n = 3) as defined by Nettleton et al. (10). The beneficial foods included dairy products, legumes/soy products, light-colored vegetables, dark-colored vegetables, fruits, whole grains, root crops, and rice and flour products, while adverse food group included milk, meats, organ meats, sugary drinks, preserved and processed foods, bread, vegetables with added oil/fats, fried (rice and flour) products, deep-fried foods, instant noodles, and seafood. However, only three food groups were classified as neutral foods (eggs, jam/honey, and soy sauce or other dips) since they were considered as non-contributors to CVD risk factors [10, 11, 22–26]. The levels of consumption were defined as 1, 2, 3, 4, and 5 from the lowest to the highest frequency. The beneficial foods were scored as +1 to +5, while the adverse foods were scored as -1 to -5 according to the levels of consumption. The total scores of the 19 food groups (beneficial and adverse foods) were finally summed up before dividing into quintiles.

### *A posteriori* dietary patterns

We further created two *a posteriori* dietary patterns that were derived using PCA. This is a well-grounded method in the study of dietary patterns [8, 9]. The two uncorrelated empirically derived dietary patterns were “vegi-fruit dietary pattern” which was characterized by high intake of vegetables and fruits and “meat-processed dietary pattern” which was characterized by high meat and processed foods intake. The PCA scores and dietary pattern components were obtained using SAS software.

### Statistical analysis

The population characteristics were summarized according to quintile levels of the three dietary patterns. The differences in the categorical variables across quintiles of dietary pattern were compared using Chi-square test while the general linear regression model (GLM) was used to compare the mean differences of the continuous indicators. We determined the distribution of our outcome indicators, of which we found that a normality assumption was sustained in all of them, except for TG that was slightly skewed. However, we performed sensitivity analyses with and without a log-transformed TG data and found no serious effect of the slight skewness. Therefore, GLM model was used for the association between the CVD risk factors and dietary patterns. Moreover, we also used the generalized linear modeling with Gaussian link function and found no difference in the coefficients. Dummy variables were created for each of the dietary patterns so as to compare the quintile levels of consumption to the reference group (quintile 1). The correlations between the outcome indicator as well as the independent variables and the outcome indicators were also assessed before using a hierarchical regression approach in building our models. Model 1 was unadjusted while model 2 was adjusted for demographic and lifestyle characteristics (i.e. sex, age, education, marital status, smoking, drinking, and physical activity) before adjusting for health characteristics (i.e. current CVD status, BMI, waist circumference, systolic and diastolic blood pressure) in model 3. We compared the adjusted coefficients of the model 3 for all the three dietary patterns to test for homogeneity using the analysis of covariance.

The scoring method for *a priori* (Taiwanese) dietary pattern was first evaluated for validity. We determined the distribution and proportion of the subjects across quintiles, and found a similar distribution of subjects across quintiles as compared with that in the previous study [11]. Therefore, the positive-negative scoring method in the present study was suitable, and food scores and ratios can be estimated using various ways depending on the research question and type of data available [27].

#### Principal component analysis

The PCA (orthogonal varimax rotation with retention of two factors), a linear combination of optimally-weighted observed variables, was used to derive the two uncorrelated patterns from the 22 food groups in the FFQ [8, 9, 28]. Generally, five different methods used in the retention of a particular number of principal components or factors included: (1) Kaiser method that allows retention of components with eigenvalues greater than 1.0, however, there are reasons given by Streiner and Norman [29] and Field et al. [30] why this is not an altogether good idea, (2) the scree test where components in the steep curve are retained before the initial point beginning the flat line trend, (3) the number of non-trivial components which involves retention of components with two or more variables loading above the cut-off-point (often 0.30), (4) *a priori* criterion where a specific number of components are set by the researcher in replication of a previous research, and (5) the percent of cumulative variance which involves retention of components that cumulatively explains the variation (usually 95%) [31–33]. We used retention of non-trivial components and percentage of variation explained. Our cut-off-point for extracting and retaining a variable within a component was set at 0.30 [34].

Retention of the two factors procedure in the statistical software enabled generation of only two uncorrelated components. Orthogonal rotation was required [22] to derive uncorrelated patterns, and this approach is not a factor analysis approach. The difference between PCA and factor analysis is in underlying causality assumption. The PCA makes no assumption about causality, it is simply a variable reduction procedure of variables accounting for the greatest variance in a set of variables, while factor analysis assumes that covariation in the observed variables is due to the existence of a latent variables (factors) exerting a causal influence on the observed variables [28]. The PCA, therefore, recognizes the uncorrelated data patterns [33]. It is often appropriate to reduce data in some set that would give a better understanding of it or simply for ease of analysis. All statistical analyses were performed using Statistical Analysis System 9.3 (SAS Institute, Cary, NC, USA).

## Results

Of the 239,362 participants, 176,397 (74%) had missing information on the potential confounders. Therefore, the analyses were restricted to 62,965 participants (30,230 females, 48%) who were 40 years and above. Two thousand nine hundred and sixty five participants (4.3%) had a history of CVD. A number of participants had abnormally high levels of LDL-C (73.8%), TC (48.9%), blood glucose (40.5%), TG (23.9%), and CRP (2.7%), while majority of the participants (82.8%) had low levels of HDL-C (data not shown).

### Dietary patterns

The theoretical minimum and maximum scores of the Taiwanese dietary pattern were -55 and 40, respectively, with a baseline mean of -4.08 and standard deviation of 4.22. The highest score indicate a diet rich in beneficial foods, which is presumed to be protective against CVD risk factors, while the lowest (negative) score indicate an adverse food-rich diet.

The PCA identified two dietary patterns. The first dietary pattern was labeled the “vegi-fruit” diet because it represented frequent intake of dark-colored vegetables, light-colored vegetables, fruits, vegetables with added oil/fat, root crops, legumes/soy products, whole grains, dairy products, and milk. The second pattern was labeled “meat-processed” diet because it reflected a frequent intake of deep-friend foods, preserved and processed foods, soy sauce or other dips, organ meats, meats, fried (rice and flour) products, sugary drinks, instant noodles, eggs, bread, and jam/honey. Only the seafood and legumes groups had component loadings greater than 0.30 in the two components but was considered under meat-processed and vegi-fruit pattern, respectively, while rice and flour products was not classified into any dietary patterns (the PCA scores are shown in Table 1). The two components had eigenvalues greater than 1.0 with cumulative percentage variance of 96.8% with the meat-processed dietary pattern accounting for the highest variation (50.4%).

**Table 1:** Principal component analysis scores for the 22 food groups of the major dietary patterns*.

Food group	Factor 1 (vegi-fruit dietary pattern)	Factor 2 (meat-processed dietary pattern)
**Dark-colored vegetables**	**0.78537**	-0.09236
**Light-colored vegetables**	**0.75728**	-0.06343
**Fruits**	**0.57054**	-0.06073
**Vegetables with added oil/fats**	**0.52376**	0.14654
**Root crops**	**0.46194**	0.22848
**Legumes/soy products**	**0.38460**	**0.31485**
**Seafood**	**0.32963**	**0.32595**
**Whole grains**	**0.32733**	0.09006
**Dairy products**	**0.32596**	0.13037
**Milk**	**0.31351**	-0.10316
**Deep-fried foods**	0.04393	**0.61730**
**Preserved and processed foods**	0.01407	**0.59198**
**Soy sauce or other dips**	-0.02304	**0.53600**
**Organ meats**	0.05869	**0.50678**
**Meats**	0.16439	**0.50382**
**Fried rice and flour products**	0.12716	**0.46757**
**Sugary drinks**	-0.07294	**0.46370**
**Instant noodles**	-0.10832	**0.41846**
**Eggs**	0.20240	**0.40557**
**Bread**	0.23219	**0.32215**
**Jam/honey**	0.17134	**0.31368**
**Rice and flour products**	0.24859	0.21069

*The values in bold indicate factors above the threshold (0.30) used in the classification of dietary patterns.

### Participant characteristics

The subjects had a similar tendency of characteristics across the quintiles of Taiwanese and vegi-fruit dietary patterns (Table 2). The proportion of males, smokers and alcohol drinkers decreased across the quintiles of Taiwanese (S1 Table) and vegi-fruit dietary patterns (S2 Table), but there was an increase in the number of participants who were engaged in physical activity (*p* < 0.001). On the contrary, the meat-processed dietary pattern (S3 Table) had an opposite trend with all the indicators being statistically differences across the quintiles at *p* < 0.001. Only CRP levels and participants with CVD was not statistically different across the quintiles of Taiwanese dietary pattern (*p* = 0.061) and vegi-fruit dietary pattern (*p* = 0.544), respectively.

**Table 2:** Characteristics of the subjects across quintiles (Q) of dietary pattern scores (*n* = 62,965)^a^.

	Taiwanese dietary pattern^b^			Vegi-fruit dietary pattern^c^^,^^d^			Meat-processed dietary pattern^c^^,^^e^		
	Q1	Q3	Q5	Q1	Q3	Q5	Q1	Q3	Q5
*n*	22 672	11 797	7 814	13 803	13 212	9575	13 423	16 747	9508
**Dietary pattern score, range**	-24 to -6	-3 to -2	1 to 19	9 to 14	17 to 18	22 to 42	11 to 17	20 to 22	26 to 56
**Sex, % male**	55.7	50.1	47.0	53.8	52.4	50.7	39.9	52.4	65.4
**Age, years (SD)**	50.4 (9.3)	52.8 (9.9)	54.2 (10.1)	52.0 (9.9)	51.7 (9.7)	52.9 (10.1)	55.2 (10.3)	51.4 (9.5)	49.1 (8.8)
**Education**									
< High school, %	23.4	28.9	29.1	32.5	24.7	23.2	39.4	23.6	17.8
High school, %	32.2	31.6	29.7	34.0	31.5	29.5	31.1	32.7	31.1
> High school, %	44.4	39.5	41.1	33.5	43.8	47.3	29.5	43.7	51.1
**Marital status**									
Never married, %	3.2	2.6	2.7	3.4	2.6	2.4	2.4	3.1	2.8
Married, %	86.6	86.1	85.2	84.0	87.1	87.3	81.8	87.1	88.9
Widows/divorced, %	10.2	11.3	12.1	12.6	10.3	10.3	15.8	9.8	8.3
**Smoking**^f^**, %**	26.3	16.3	10.0	28.1	18.1	13.6	11.9	19.2	31.5
**drinking** ^f^**, %**	22.9	17.0	11.7	21.7	18.1	16.5	11.9	18.9	26.9
**Physical activity**^f^**, %**	57.5	65.8	75.6	46.1	67.5	79.1	62.8	64.6	61.6
**Cardiovascular disease, %**	3.9	4.5	5.0	4.1	4.3	4.6	5.4	4.1	3.5
**Body mass index, kg/m**^**2**^ **(SD)**	23.9 (3.3)	23.7 (3.1)	23.4 (3.2)	23.9 (3.3)	23.8 (3.2)	23.7 (3.1)	23.6 (3.2)	23.8 (3.2)	24.2 (3.3)
**Waist circumference, cm (SD)**	80.1 (9.9)	79.1 (9.4)	78.3 (9.3)	80.1 (10.0)	79.4 (9.5)	78.9 (9.4)	78.2 (9.4)	79.3 (9.5)	81.5 (9.9)
**Systolic blood pressure, mm Hg (SD)**	122.2 (18.7)	123.9 (19.5)	124.5 (20.1)	123.9 (19.7)	122.7 (18.9)	123.4 (19.4)	125.7 (20.7)	122.5 (19.0)	121.8 (17.9)
**Diastolic blood pressure, mm Hg (SD)**	73.7 (11.7)	74.2 (11.7)	73.9 (11.9)	74.2 (12.0)	73.8 (11.6)	73.8 (11.7)	70.9 (19.0)	70.5 (18.9)	71.2 (18.3)
**Blood lipids**									
Triacylglycerol, mmol/l (SD)	1.4 (0.7)	1.3 (0.7)	1.3 (0.7)	1.4 (0.8)	1.4 (0.7)	1.3(0.7)	1.3 (0.7)	1.3 (0.7)	1.4 (0.8)
Total cholesterol, mmol/l (SD)	5.2 (0.9)	5.1 (0.9)	5.0 (0.9)	5.2 (0.9)	5.3 (0.9)	5.1(0.9)	5.2 (0.9)	5.1 (0.9)	5.2 (0.9)
LDL-C, mmol/l (SD)	3.2 (0.8)	3.1 (0.8)	2.9 (0.8)	3.1 (0.8)	3.1 (0.8)	3.1(0.8)	3.1 (0.8)	3.0 (0.8)	3.2 (0.8)
HDL-C, mmol/l (SD)	1.4 (0.4)	1.5 (0.4)	1.5 (0.4)	1.4 (0.4)	1.4 (0.4)	1.5 (0.4)	1.5 (0.4)	1.5 (0.4)	1.4 (0.4)
**C-reactive protein, nmol/l (SD)**	23.8 (45.3)	23.9 (50.5)	22.1 (42.8)	25.9 (50.8)	24.1 (54.8)	21.8 (38.5)	24.7 (54.7)	23.6 (48.8)	23.4 (40.1)
**Fasting glucose, mmol/l (SD)**	3.9 (1.2)	3.8 (1.1)	3.6 (1.0)	3.9 (1.2)	3.8 (1.1)	3.7 (1.1)	3.7 (1.1)	3.8 (1.1)	4.0 (1.2)

^a^Data are expressed as range, %, or mean (SD). General linear regression was used to test for trend with dietary pattern treated as continuous explanatory variable, while χ^2^ test was employed for categorical variables across all quintile levels of dietary patterns.

^b^All variables are significant at *p* < 0.001, except for CRP significant at *p* = 0.003 and diastolic blood pressure not significant at *p* = 0.355.

^c^ Vegi-fruit and meat-processed dietary patterns are identified by principal component analysis, centered on 0 with a SD of 1.0.

^d^All variables were significant at *p* < 0.001, except for systolic blood pressure (*p* = 0.834), diastolic blood pressure (*p* = 0.729), and CVD (*p* = 0.544).

^e^All variables significant at *p* < 0.001.

^**f**^Smoking: ≥ 1–3 times/ week; drinking: ≥ 1–2 times/week; physical activity: ≥ 1–2 hours/week.

The correlation analysis of the outcome variables indicated that blood TG, TC, LDL-C, and fasting glucose levels were positively correlated to each other (*p* < 0.001) (S4 Table). Additionally, blood TC and HDL-C levels were positively correlated (*p* < 0.001). However, there was a negative correlation between blood HDL-C levels and blood TG, LDL-C, CRP, and fasting glucose levels (*p* < 0.001).

The unadjusted association between demographic data, lifestyle, health characteristics and CVD risk factors are presented in Table 3 (see also the correlation coefficient in S5 Table). Male gender was positively associated with blood TG, LDL-C, CRP, and fasting glucose levels, but negatively associated with TC and HDL-C levels compared with female gender (*p* < 0.001). The lowest education level (< high school) was positively associated with blood TG, TC, LDL-C, HDL-C, and CRP levels, but negatively associated with fasting glucose levels as compared to the highest education level (*p* < 0.001). High physical activity was positively associated with blood TC (*p* < 0.01), LDL-C, and HDL-C levels, but negatively associated with blood TG, CRP, and fasting glucose levels as compared to sedentary activity (*p* < 0.001). Age, BMI, and blood pressure were also positively associated with blood TG, TC, LDL-C, CRP, and fasting glucose levels (*p* < 0.001). Smoking, drinking, CVD, BMI, and diastolic blood pressure had a negative association with HDL-C levels (*p* < 0.001).

**Table 3:** Unadjusted linear regression coefficients (95% confidence interval) for respondents’ characteristics and cardiovascular disease risk factors among the adults aged 40 years and above in Taiwan.

	Regression coefficient (95% confidence interval), *n* = 62,965					
	Triacylglycerol	Total cholesterol	LDL-C	HDL-C	C-reactive protein	Fasting glucose
**Sex (reference: female)**	27.32 (26.34, 28.30)^3^	-1.02 (-1.57, -0.48)^3^	5.58 (5.10, 6.07)^3^	-12.08 (-12.3, -11.8)^3^	0.02 (0.01, 0.03)^3^	0.81 (0.79, 0.87)^3^
**Age**	0.44 (0.39, 0.49)^3^	0.39 (0.36, 0.42)^3^	0.32 (0.29, 0.34)^3^	-0.009 (-0.02, 0.003)	0.004 (0.003, 0.004)^3^	0.006 (0.005, 0.007)^3^
**Education (reference: > high school)**						
< High school	4.62 (3.37, 5.86)^3^	5.71 (5.04, 6.39)^3^	2.53 (1.92, 3.14)^3^	2.25 (1.95, 2.55)^3^	0.07 (0.06, 0.08)^3^	-0.06 (-0.09, -0.04)^3^
High school	-1.85 (-3.02, -0.67)^2^	0.66 (0.02, 1.30)^1^	-0.45 (-1.02, 0.13)	1.47 (1.19, 1.76)^3^	0.01 (-0.001, 0.02)	-0.09 (-0.11, -0.06)^3^
**Marital status (reference: married)**						
Never married	-17.09 (-20.1, -14.1)^3^	-1.49 (-3.13, 0.15)	-3.09 (-4.56, -1.62)^3^	5.03 (4.31, 5.75)^3^	-0.02 (-0.05, -0.002)^1^	-0.33 (-0.39, -0.28)^3^
Widow/divorced	0.26 (-1.35, 1.88)	4.61 (3.74, 5.48)^3^	1.02 (0.23, 1.81)^1^	3.55 (3.16, 3.93)^3^	0.03 (0.02, 0.05)^3^	-0.17 (-0.20, -0.14)^3^
**Smoking (reference: non-smoker)**	23.86 (22.61, 25.11)^3^	-0.88 (-1.56, -0.19)^1^	2.03 (1.41, 2.64)^3^	-7.67 (-7.79, -7.38)^3^	0.03 (0.02, 0.04)^3^	0.57 (0.55, 0.59)^3^
**Drinking (reference: non-drinker)**	17.64 (16.36, 18.92)^3^	0.48 (-0.22, 1.18)	-0.95 (-1.58, -0.32)^2^	-2.09 (-2.40, -1.78)^3^	-0.002 (0.01, 0.01)	0.15 (0.13, 0.17)^3^
**Physical activity (reference: sedentary)**	-3.41 (-4.46, -2.37)^3^	0.85 (0.28, 1.42)^2^	0.91 (0.40, 1.42)^3^	0.63 (0.38, 0.88)^3^	-0.03 (-0.03, -0.02)^3^	-0.04 (-0.06, -0.02)^3^
**Cardiovascular disease (reference: no CVD)**	9.27 (6.79, 11.74)^3^	0.59 (-0.75, 1.94)	0.50 (-0.71, 1.71)	-1.76 (-2.36, -1.16)^3^	0.06 (0.04, 0.08)^3^	0.12 (0.08, 0.16)^3^
**Body mass index**	6.67 (6.52, 6.82)^3^	1.24 (1.16, 1.32)^3^	1.58 (1.51, 1.66)^3^	-1.68 (-1.71, -1.64)^3^	0.01 (0.01, 0.01)^3^	0.13 (0.13, 0.14)^3^
**Waist circumference**	2.61 (2.56, 2.66)^3^	0.37 (0.34, 0.40)^3^	0.58 (0.55, 0.60)^3^	-0.73 (-0.74, -0.72)^3^	0.005 (0.004, 0.005)^3^	0.06 (0.05, 0.06)
**Systolic blood pressure**	0.61 (0.58, 0.63)^3^	0.23 (0.22, 0.24)^3^	0.18 (0.16, 0.19)^3^	-0.07 (-0.07, -0.06)	0.001 (0.001, 0.002)^3^	0.01 (0.01, 0.01)^3^
**Diastolic blood pressure**	1.21 (1.17, 1.26)^3^	0.33 (0.31, 0.35)^3^	0.28 (0.26, 0.30)^3^	-0.20 (-0.21, -0.19)^3^	0.002 (0.002, 0.002)^3^	0.02 (0.02, 0.02)^3^

^1^*p <* 0.05.

^2^*p* < 0.01.

^3^*p <* 0.001.

### Relationship between dietary patterns and CVD risk factors

The unadjusted and adjusted relationships of the three between dietary patterns and CVD risk factors were presented in Table 4. Before adjustment (model 1), the highest level of Taiwanese dietary pattern (Q5) was negatively associated with blood TG (*β* = -6.39; CI: -8.04, -4.74), TC (*β* = -6.57; CI: -7.47, -5.68), LDL-C (*β* = -6.35; CI: -7.16, -5.55), CRP (*β* = -0.017; CI: -0.03, -0.005), and fasting glucose (*β* = -0.22; CI: -0.25, -0.19). However, after a multivariable adjustment of demographic and lifestyle characteristics (models 2) and further adjustment for health characteristics in model 3, there was no association between Taiwanese dietary pattern and blood TG levels. Only blood HDL-C levels (*β* = 1.07; CI: 0.67, 1.46) was positive before adjustment (*p* < 0.001), but became negative (*β* = -1.13; CI: -1.48, -0.78) in model 3. Moreover, the pattern was negatively associated with blood CRP levels only at Q5 level of consumption in all models.

**Table 4:** Multivariable linear regression coefficients (95% confidence interval) for the association between dietary patterns and cardiovascular disease risk factors among the adults aged 40 years and above in Taiwan.

	Regression coefficient (95% confidence interval), *n* = 62,965					
	Triacylglycerol	Total cholesterol	LDL-C	HDL-C	C-reactive protein	Fasting glucose
**Taiwanese dietary pattern**						
Model 1^a^ (reference: Q1)						
Q2	-2.09 (-3.49, -0.70)^2^	-1.42 (-2.18, -0.66)^3^	-1.64 (-2.33, -0.96)^3^	0.64 (0.31, 0.98)^3^	-0.001 (-0.012, 0.01)	-0.08 (-0.11, -0.06)^3^
Q3	-3.96 (-5.38, -2.53)^3^	-1.88 (-2.66, -1.11)^3^	-2.11 (-2.81, -1.42)^3^	1.02 (0.68, 1.36)^3^	-0.001 (-0.01, 0.012)	-0.13 (-0.15, -0.10)^3^
Q4	-3.52 (-5.15, -1.90)^3^	-3.37 (-4.25, -2.49)^3^	-3.53 (-4.32, -2.74)^3^	0.86 (0.47, 1.25)^3^	-0.001 (-0.012, 0.013)	-0.15 (-0.18, -0.12)^3^
Q5	-6.39 (-8.04, -4.74)^3^	-6.57 (-7.47, -5.68)^3^	-6.35 (-7.16, -5.55)^3^	1.07 (0.67, 1.46)^3^	-0.017 (-0.03, -0.005)^3^	-0.22 (-0.25, -0.19)^3^
Model 2^b^ (reference: Q1)						
Q2	-0.51 (-0.87, 0.86)	-2.08 (-2.83, -1.32)^3^	-2.01 (-2.69, -1.34)^3^	0.04 (-0.27, 0.35)	-0.003 (-0.014, 0.008)	-0.05 (-0.07, 0.02)^3^
Q3	-2.00 (-3.40, -0.61)^2^	-3.06 (-3.83, -2.28)^3^	-2.85 (-3.55, -2.16)^3^	0.19 (-0.12, 0.51)	-0.004 (-0.015, 0.007)	-0.08 (-0.11, -0.06)^3^
Q4	-1.16 (-2.75, 0.43)	-4.71 (-5.60, -3.83)^3^	-4.44 (-5.24, -3.65)^3^	-0.04 (-0.40, 0.32)	-0.005 (-0.017, 0.008)	-0.10 (-0.13, -0.08)^3^
Q5	-2.73 (-4.36, -1.10)^2^	-8.43 (-9.33, -7.52)^3^	-7.61 (-8.42, -6.79)^3^	-0.27 (-0.64, 0.10)	-0.024 (-0.037, -0.011)^3^	-0.15 (-0.18, -0.13)^3^
Model 3^c^ (reference: Q1)						
Q2	0.65 (-0.63, 1.93)	-1.83 (-2.60, -1.10)^3^	-1.77 (-2.44, -1.10)^3^	-0.21 (-0.50, 0.08)	-0.001 (-0.012, 0.009)	-0.03 (-0.05, -0.01)^1^
Q3	-0.20 (-1.51, 1.12)	-2.78 (-3.55, -2.02)^3^	-2.49 (-3.17, -1.80)^3^	-0.26 (-0.56, 0.04)	-0.003 (-0.011, 0.011)	-0.05 (-0.07, 0.03)^3^
Q4	0.79 (-0.71, 2.28)	-4.39 (-5.27, -3.52)^3^	-4.06 (-4.84, -3.27)^3^	-0.50 (-0.84, -0.16)^1^	-0.001 (-0.014, 0.011)	-0.07 (-0.09, -0.04)^3^
Q5	0.83 (-0.70, 2.37)	-7.83 (-8.73, -6.93)^3^	-6.86 (-7.66, -6.06)^3^	-1.13 (-1.48, -0.78)^3^	-0.017 (-0.03, -0.004)^2^	-0.09 (-0.11, -0.06)^3^
**Vegi-fruit dietary pattern**						
Model 1^a^ (reference: Q1)						
Q2	-4.12 (-5.65, -2.59)^3^	-0.67 (-1.51, 0.16)	-0.48 (-1.22, 0.27)	0.63 (0.26, 1.00)^3^	-0.030 (-0.042, -0.019)^3^	-0.07 (-0.09, -0.04)^3^
Q3	-5.60 (-7.13, -4.07)^3^	-0.89 (-1.72, -0.06)^1^	-0.73 (-1.48, 0.02)	0.96 (0.59, 1.33)^3^	-0.019 (-0.031, -0.007)^2^	-0.10 (-0.12, -0.07)^3^
Q4	-7.16 (-8.69, -5.63)^3^	-1.80 (-2.63, -0.97)^3^	-1.65 (-2.40, -0.91)^3^	1.28 (0.92, 1.65)^3^	-0.036 (-0.048, -0.024)^3^	-0.14 (-0.17, -0.12)^3^
Q5	-8.71 (-10.38, -7.04)^3^	-2.93 (-3.83, -2.02)^3^	-2.86 (-3.67, -2.04)^3^	1.67 (1.27, 2.07)^3^	-0.043 (-0.056, -0.031)^3^	-0.19 (-0.22, -0.16)^3^
Model 2^b^ (reference: Q1)						
Q2	-1.40 (-2.89, 0.10)	-0.57 (-1.40, 0.27)	-0.27 (-1.01, 0.48)	-0.02 (-0.36, 0.32)	-0.022 (-0.033, -0.010)^3^	-0.02 (-0.04, 0.01)
Q3	-2.48 (-3.99, -0.97)^2^	-0.88 (-1.72, -0.04)^1^	-0.70 (-1.45, 0.05)	0.31 (-0.03, 0.65)	-0.008 (-0.020, 0.004)	-0.05 (-0.07, -0.02)^3^
Q4	-3.21 (-4.73, -1.69)^3^	-1.86 (-2.70, -1.01)^3^	-1.63 (-2.38, -0.87)^3^	0.41 (0.07, 0.76)^1^	-0.023 (-0.035, -0.011)^3^	-0.08 (-0.10, -0.05)^3^
Q5	-4.37 (-6.04, -2.70)^3^	-3.45 (-4.38, -2.52)^3^	-3.16 (-3.99, -2.32)^3^	0.58 (0.20, 0.95)^2^	-0.032 (-0.045, -0.019)^3^	-0.12 (-0.15, -0.09)^3^
Model 3^c^ (reference: Q1)						
Q2	-1.07 (-2.47, 0.34)	-0.47 (-1.30, 0.35)	-0.20 (-0.94, 0.54)	-0.06 (-0.38, 0.26)	-0.021 (-0.033, -0.009)^3^	-0.01 (-0.04, 0.01)
Q3	-1.89 (-3.31, -0.47)^2^	-0.73 (-1.56, -0.10)	-0.59 (-1.34, 0.15)	0.24 (-0.08, 0.56)	-0.007 (-0.019, 0.005)	-0.04 (-0.06, -0.01)^2^
Q4	-1.93 (-3.56, -0.50)^2^	-1.57 (-2.41, -0.73)^3^	-1.37 (-2.12, -0.62)^3^	0.19 (-0.13, 0.51)	-0.021 (-0.034, -0.009)^3^	-0.06 (-0.08, -0.03)^3^
Q5	-2.96 (-4.53, -1.39)^3^	-3.16 (-4.08, -2.24)^3^	-2.89 (-3.72, -2.07)^3^	0.32 (-0.03, 0.68)	-0.030 (-0.043, -0.017)^3^	-0.09 (-0.12, -0.07)^3^
**Meat-processed dietary pattern**						
Model 1^a^ (reference: Q1)						
Q2	-2.30 (-3.89, -0.71)^2^	1.36 (-0.51, 1.22)	0.84 (0.06, 1.62)^1^	-0.004 (-0.39, 0.38)	-0.013 (-0.026, -0.001)^1^	0.01 (-0.02, 0.04)
Q3	-0.04 (-1.49, 1.42)	0.22 (-0.57, 1.01)	1.09 (0.38, 1.80)^2^	-0.86 (-1.21, -0.51)^3^	-0.011 (-0.022, -0.001)^1^	0.07 (0.05, 0.10)^3^
Q4	4.78 (3.20, 6.38)^3^	1.21 (0.35, 2.08)^2^	2.11 (1.34, 2.89)^3^	-1.86 (-2.24, -1.47)^3^	-0.022 (-0.034, -0.009)^3^	0.17 (0.14, 0.20)^3^
Q5	9.11 (7.43, 10.80)^3^	2.06 (1.14, 2.97)^3^	3.58 (2.76, 4.40)^3^	-3.35 (-3.76, -2.95)^3^	-0.014 (-0.027, -0.001)^1^	0.29 (0.26, 0.32)^3^
Model 2^b^ (reference: Q1)						
Q2	-2.60 (-4.15, -1.05)^3^	1.53 (0.67, 2.39)^3^	1.41 (0.64, 2.18)^3^	0.65 (0.30, 1.01)^3^	-0.002 (-0.015, 0.010)	-0.02 (-0.04, 0.01)
Q3	-1.49 (-2.92, -0.05)^1^	2.14 (1.34, 2.93)^3^	1.98 (1.27, 2.70)^3^	0.46 (0.13, 0.78)^2^	0.005 (-0.007, 0.016)	0.01 (-0.01, 0.04)
Q4	1.58 (-0.01, 3.15)	3.72 (2.84, 4.60)^3^	3.09 (2.30, 3.88)^3^	0.33 (-0.04, 0.68)	-0.003 (-0.015, 0.010)	0.06 (0.03, 0.08)^3^
Q5	3.79 (2.10, 5.49)^3^	5.12 (4.17, 6.06)^3^	4.73 (3.88, 5.58)^3^	-0.38 (-0.76, 0.01)	0.006 (-0.007, 0.020)	0.13 (0.10, 0.16)^3^
Model 3^c^ (reference: Q1)						
Q2	-3.20 (-4.65, -1.74)^3^	1.45 (0.59, 2.30)^3^	1.27 (0.50, 2.03)^3^	0.84 (0.50, 1.17)^3^	-0.003 (-0.016, 0.009)	-0.03 (-0.05, -0.004)^1^
Q3	-3.32 (-4.67, -1.97)^3^	1.85 (1.06, 2.65)^3^	1.59 (0.88, 2.30)^3^	0.93 (0.63, 1.24)^3^	0.001 (-0.010, 0.012)	-0.03 (-0.05, -0.003)^1^
Q4	-1.18 (-2.67, 0.31)	3.28 (2.40, 4.15)^3^	2.50 (1.72, 3.28)^3^	1.01 (0.68, 1.35)^3^	-0.008 (-0.020, 0.005)	0.003 (-0.02, 0.03)
Q5	-1.36 (-2.96, 0.23)	4.23 (3.29, 5.17)^3^	3.63 (2.79, 4.47)^3^	0.87 (0.51, 1.23)^3^	-0.003 (-0.017, 0.010)	0.03 (0.005, 0.06)^1^

^a^Unadjusted model.

^b^Adjusted for sex, age, education, marital status, smoking, drinking, and physical activity.

^c^Adjusted for the variables in model 2 plus cardiovascular disease, body mass index, waist circumference, systolic blood pressure, and diastolic blood pressure.

^1^*p <* 0.05.

^2^*p* < 0.01.

^3^*p <* 0.001.

The vegi-fruit dietary pattern (Q5) was also negatively associated with TG (*β* = -2.96; CI: -4.53, -1.39), TC (*β* = -3.16; CI: -4.08, -2.24), LDL-C (*β* = -2.89; CI: -3.72, -2.07), CRP (*β* = -0.030; CI: -0.043, -0.017), and fasting glucose (*β* = -0.09; CI: -0.12, -0.07) levels in model 3 at *p* < 0.001 but positively associated with blood HDL-C levels before adjustment (*β* = 1.67; CI: 1.27, 2.07) and no association in model 3. On the contrary, after adjustment of all confounders the meat-processed dietary pattern (Q5) was positively related with TC (*β* = 4.23; CI: 3.29, 5.17), LDL-C (*β* = 3.63; CI: 2.79, 4.47), HDL-C (*β* = 0.87; CI: 0.51, 1.23) and fasting glucose (*β* = 0.03; CI: 0.005, 0.06) but negatively related with TG (*β* = -3.32; CI: -4.67, -1.97) in Q3 levels while the association with CRP was attenuated.

The test for homogeneity of the dietary patterns indicated that the patterns were statistically different from one another after adjusting for all the potential confounders (Table 5). The Taiwanese and vegi-fruit dietary patterns were negatively associated with all the CVD risk factors (*p* < 0.01); except for the relationship between Taiwanese dietary pattern and blood TG levels, and vegi-fruit dietary pattern and blood HDL-C levels. However, blood TC, LDL-C, HDL-C, and fasting glucose levels were positively associated with meat-processed dietary pattern (*p* < 0.001). Moreover, the differences between the adjusted regression coefficients of these patterns were statistically significant at *p* < 0.001.

**Table 5:** Comparison of the regression coefficients (95% confidence interval) for the association between dietary patterns and cardiovascular disease risk factors among the adults aged 40 years and above in Taiwan^a^.

	Regression coefficient *β* (95% confidence interval), *n* = 62,965		
	Taiwanese dietary pattern	Vegi-fruit dietary pattern	Meat-processed dietary pattern
**Triacylglycerol**	0.07 (-0.04, 1.78)	-0.26 (-0.38, -0.14)^b^^,^^3^	-0.03 (-0.13, 0.08)^b^^,^^c^
**Total cholesterol**	-0.63 (-0.70, -0.57)^3^	-0.29 (-0.36, -0.22)^b^^,^^3^	0.31 (0.25, 0.38)^b^^,^^c^^,^^3^
**LDL-C**	-0.55 (-0.61, -0.50)^3^	-0.27 (-0.33, -0.21)^b^^,^^3^	0.26 (0.20, 0.31)^b^^,^^c^^,^^3^
**HDL-C**	-0.09 (-0.12, -0.07)^3^	0.03 (-0.002, 0.05)^b^	0.06 (0.04, 0.09)^b^^,^^c^^,^^3^
**C-reactive protein**	-0.001 (-0.002, -0.0002)^1^	-0.002 (-0.003, -0.001)^b^^,^^3^	-0.0004 (-0.001, 0.0005)^b^^,^^c^
**Fasting glucose**	-0.007 (-0.009, -0.005)^3^	-0.008 (-0.01, -0.006)^b^^,^^3^	0.003 (0.001, 0.005)^b^^,^^c^^,^^3^

^a^ Continuous scores were used to determine the association between dietary patterns and cardiovascular disease risk factors after adjusting for sex, age, education, marital status, smoking, drinking, physical activity, cardiovascular disease, body mass index, waist circumference, systolic blood pressure, and diastolic blood pressure.

^b^ Significant difference compared with Taiwanese dietary pattern at *p* < 0.001.

^c^ Significant difference compared with vegi-fruit dietary pattern at *p* < 0.001.

^1^*p <* 0.05.

^2^*p* < 0.01.

^3^*p <* 0.001.

## Discussion

In this cross-sectional study of 62,965 middle-aged and elderly adults in Taiwan, we derived three dietary patterns using two different approaches (*a priori* and *a posteriori*) and found a significant association with CVD risk factors. Our findings suggests that high consumption of Taiwanese dietary pattern and vegi-fruit dietary pattern may reduce CVD risk factors, while high intake of the meat-processed dietary pattern may increase the risk factors of CVD. The differences in demographic, lifestyle and health characteristics could not explain these associations.

This study demonstrated that positive-negative scoring approach can be utilized in the study of the association between *a priori* dietary pattern and CVD risk factors; supporting authors who noted that scores and ratios can be estimated using various ways, depending on the research question and type of data available [27]. In addition, there are several parallels between our PCA-derived dietary patterns and those already identified and established in previous literature on different populations. In fact, the vegi-fruit dietary pattern in our study is comparable to the vegetable-rich pattern, labeled as ‘vegetables’ or ‘prudent’, while the meat-processed dietary patterns is similar to patterns high in meat, fast foods and sugary foods referred to as the ‘Western’ pattern [35–37].

Our study found that the highest quintile of consumption (Q5) of Taiwanese diet reduces TC, LDL-C, HDL-C, CRP and fasting glucose levels in comparison to the lowest quintile (Q1) with an effect size of -7.83, -6.86, -1.13, -0.017 and -0.09, respectively. The Taiwanese dietary pattern is similar to the “Healthy” dietary pattern that was found to be associated with lower CVD risk factors [10]. Several other studies have also shown that diets characterized by a high consumption of plant-based foods and a low intake of meats and processed foods are favorably associated with CVD risk factors [12, 38–40]. Founded on these studies among other studies that assessed nutrients or individual food items we created *a priori* “healthy”, vegetable-centered dietary pattern [41–46].

This study found that the highest intake (Q5) of the vegi-fruit diet reduces TG, TC, LDL-C, CRP and fasting glucose levels with an effect size of -2.96, -3.16, -2.89, -0.03 and -0.09, respectively, as compared to the lowest level of consumption (Q1). However, there was a positive association between vegi-fruit dietary pattern and HDL-C levels before adjusting for the potential confounders; but after the adjustment of demographic, lifestyle and health characteristics, the difference was explained. The associations found between vegi-fruit dietary pattern and CVD risk factors were generally in agreement with studies that have shown a protective effect of vegetable-rich pattern on the risk factors of metabolic-related diseases among the Asian populations [23, 47–51]. Similar food consumption such as whole grains and soy products, potato, fruits, and vegetables, and other traditional Chinese diet including rice, high vegetable intake, and low animal products have been associated with to lower CVD risk factors among Asian populations [24, 25, 49, 51–53].

Our findings suggested that high consumption (Q5) of meat-processed dietary pattern was associated with high TC, LDL-C, HDL-C, and fasting glucose levels as compared to the lowest quintile of consumption with a higher mean value of 4.23, 3.63, 0.87 and 0.03, respectively. Authors have found that high intake of fast food, sugary drinks and meat and processed foods was significantly associated with CVD risk factors [49, 52, 54–58]. Similarly, other authors found that “Western” dietary pattern increases CVD risk factors as found in our study [59, 60]. Moreover, individual food group analysis have shown that meats and fried foods are adversely associated with CVD risk factors [35]. In this study, we found that the meat-processed dietary pattern was negatively associated with CRP levels in the crude model; however, the difference was attenuated after adjusting for demographic and lifestyle characteristics. This was contrary to another study which found that high intake of meat and pasta diet was positively associated to high CRP levels [61]. Nevertheless, other studies have also revealed a marginal meat diet association with CRP, especially among women [62], and no association among both men and women [25].

High consumption of both the Taiwanese and the vegi-fruit dietary patterns lowered blood lipid levels, while high consumption of the meat-processed dietary pattern increased blood lipid levels. Previous studies have shown that intake of the diet rich in fruits, vegetables and whole grain increased fiber intake and consequently lowered blood lipid levels [63, 64], and was also associated with reduced oxidative stress and inflammation [65]. However, low fiber intake as observed in the meat dietary pattern increased the absorption of saturated fats, usually found in meat and animal products, due to prolonged gastrointestinal tract transit time. The saturated fats in red meat were related with increased risk of CVD, and it is possibly because of elevated blood cholesterol level [66]. Moreover, high intake of meats also increased insulin resistance which could be mediated by saturated fats [67]. Therefore, in managing blood lipid levels, high fiber in the diet would result in absorption of saturated fats and consequently eliminating it from the body. In addition, high fiber consumption improved insulin sensitivity, further regulated blood glucose level [68], and decreased the inflammatory marker (CRP) [69]. These mechanisms would possibly explain the different associations between three dietary patterns and CVD risk factors.

### Strengths, limitations and future study

Our study had strengths and limitations. The strengths included extensive information and analyses of the Taiwanese diet based on a standardized and validated FFQ and the large sample size which may be representative of the Taiwanese middle-aged and elderly adults. Thirdly, to the best of our knowledge, this is the first study in Taiwan to study the relationship between dietary patterns and CVD risk factors using a large sample size. Fourthly, two different approaches used in the development of dietary patterns give this study strength. Since diet is a complex exposure, it is always appropriate to study its association with risk factors of a disease using multiple approaches [70]. Fifthly, the PCA approach is also advantageous because it attenuates data redundancy (i.e. given the orthogonal components), eliminates problems of multicollinearity (model over-fitting) between dietary items, increases statistical power in detecting association, and reduces the complexity of high dimensionality found in data by reducing the number of dimensions without much loss of information. The use of PCA approach to generate dietary patterns enabled a strong internal validity in this study. However, the main weaknesses of PCA are related to the problem of reproducibility over time and among different studies due to many arbitrary decisions during the process of pattern derivation, and inability to link the outcome with a single food item [70].

In addition, several design and methodological limitations in our study should also be noted. Firstly, the FFQ used during screening provided only the information on an estimate of habitual food intake but not nutrient consumption. Secondly, our scoring technique may also have weaknesses since it differ from the techniques in other dietary patterns such as Mediterranean diet [71] and Alternate Healthy Eating Index [72]. Future studies should consider developing a tool that would collect a wide range of food item, nutrients composition and type of cooking oil/fat. The use of a different scoring approach should also be considered. Thirdly, individuals who might have visited the health screening center may have been health-conscious and may have changed their dietary habits. Furthermore, there could have been self-reporting bias associated with the self-administered questionnaire. To overcome these challenges we performed a sensitivity analysis by retaining different number of components and comparing the extracted component loadings. The result from our sensitivity analyses indicated that the dietary patterns were robust. Fourthly, the cross-sectional study design only showed a picture of one point-in-time, and hence this study presented a temporal causal relationship between dietary patterns and CVD risk factors. The possibility of a reverse causation also exists. Future study should consider cohort or randomized trial designs to establish a causal relationship between dietary patterns and CVD risk factors in Taiwan. Finally, since Taiwan has 99% national health insurance coverage and unlimited hospital and doctor visit, there may be a selection bias and thus unrepresentativeness of the study population. Future studies should consider subjects in different facilities across different regions in Taiwan.

## Conclusions

In conclusion, our findings support the association between dietary patterns and CVD risk factors. Both Taiwanese and vegi-fruit dietary patterns were reflective of foods high in vegetables, fruits, legumes and soy products, and whole grains foods, and were negatively associated with CVD risk factors; while the meat-processed dietary pattern was positively associated with CVD risk factors. Hence, diet could be beneficial in the management of CVD risk factors. *A priori* and *a posteriori* approaches used to derive dietary patterns are also applicable to the study of the dietary patterns and CVD risk factors despite their conceptual differences. Public health disseminating the general dietary recommendations is likely to make a positive impact on health status of Taiwan population.

## Supporting Information

S1 Table Characteristics of the subjects across quintiles (Q) of *a priori* dietary pattern scores. (PDF)

S2 Table Characteristics of the subjects across quintiles (Q) of vegi-fruit dietary pattern scores. (PDF)

S3 Table Characteristics of the subjects across quintiles (Q) of meat-processed dietary pattern scores. (PDF)

S4 Table Pearson’s correlation coefficients (*r*) of cardiovascular disease risk factors. (PDF)

S5 Table Pearson’s correlation coefficients (*r*) between demographic data, lifestyle, or health characteristics and cardiovascular disease risk factors. (PDF)
