# Supplementary material for: Association between Dietary Patterns and Cardiovascular Risk Factors among Middle-Aged and Elderly Adults in Taiwan: A Population-Based Study from 2003 to 2012
Source: PLoS One. 2016 Jul 1;11(7):e0157745. doi: 10.1371/journal.pone.0157745 (PMC4930186; doi:10.1371/journal.pone.0157745)
Supplement: S1 Table — (PDF) [file pone.0157745.s001.pdf]

Supporting Information

S1 Table. Characteristics of the subjects across quintiles (Q) of Taiwanese dietary pattern.[1]

	Taiwanese dietary pattern					
	**Q1**	**Q2**	**Q3**	**Q4**	**Q5**	***P* for trend**
n	22 672	15 557	11 797	8 125	7 814	-
**Dietary pattern score, range**	-24 to -6	-5 to -4	-3 to -2	-1 to 0	1 to 19	-
**Sex, % male**	55.7	51.4	50.1	49.7	47.0	< 0.001
**Age, y (SD)**	50.4 (9.3)	51.7 (9.7)	52.8 (9.9)	53.2 (10.0)	54.2 (10.1)	< 0.001
**Education**						< 0.001
**< High school, %**	23.4	25.9	28.9	28.5	29.1	
**High school, %**	32.2	32.9	31.6	32.0	29.7	
**> High school, %**	44.4	41.2	39.5	39.5	41.1	
**Marital status**						< 0.001
**Never married, %**	3.2	2.9	2.6	2.3	2.7	
**Married, %**	86.6	86.4	86.1	86.5	85.2	
**Widows/divorced, %**	10.2	10.7	11.3	11.2	12.1	
**Smoking, %**	26.3	19.5	16.3	14.8	10.0	< 0.001
**Drinking, %**	22.9	18.9	17.0	15.3	11.7	< 0.001
**Physical activity, %**	57.5	63.1	65.8	69.0	75.6	< 0.001
**Cardiovascular disease, %**	3.9	4.1	4.5	4.7	5.0	< 0.001
**Body mass index, kg/m^2 (SD)**	23.9 (3.3)	23.8 (3.1)	23.7 (3.1)	23.7 (3.2)	23.4 (3.2)	< 0.001
**Waist circumference, cm**	80.1 (9.9)	79.5 (9.5)	79.1 (9.4)	79.1 (9.4)	78.3 (9.3)	< 0.001
**Systolic blood pressure, mm Hg (SD)**	122.2 (18.7)	122.6 (18.9)	123.9 (19.5)	124.0 (19.6)	124.5 (20.1)	< 0.001
**Diastolic blood pressure, mm Hg (SD)**	73.7 (11.7)	73.5 (11.5)	74.2 (11.7)	73.9 (11.8)	73.9 (11.9)	0.017
**Blood lipids**						
**Triacylglycerol, mmol/L (SD)**	1.4 (0.7)	1.4 (0.7)	1.3 (0.7)	1.4 (0.7)	1.3 (0.7)	< 0.001
**Total cholesterol, mmol/L (SD)**	5.2 (0.9)	5.2 (0.9)	5.1 (0.9)	5.1 (0.9)	5.0 (0.9)	< 0.001
**LDL-C, mmol/L (SD)**	3.2 (0.8)	3.1 (0.8)	3.1 (0.8)	3.1 (0.8)	2.9 (0.8)	< 0.001
**HDL-C, mmol/L (SD)**	1.4 (0.4)	1.4 (0.4)	1.5 (0.4)	1.4 (0.4)	1.5 (0.4)	< 0.001
**C-reactive protein, nmol/L (SD)**	23.8 (45.3)	23.7 (49.2)	23.9 (50.5)	23.8 (46.0)	22.1 (42.8)	0.061
**Fasting glucose, mmol/L (SD)**	3.9 (1.2)	3.8 (1.1)	3.8 (1.1)	3.7 (1.1)	3.6 (1.0)	< 0.001

[1]Data are expressed as range, %, or mean (SD). General linear regression was used to test for trend with dietary pattern treated as continuous explanatory variable, while χ^2 test was employed for categorical variables across all quintile levels of dietary patterns. Q1: the lowest dietary pattern score of Taiwanese dietary pattern; Q5: the highest dietary score of Taiwanese dietary pattern; smoking: ≥ 1-3 times/week; drinking: ≥ 1-2 times/week; physical activity: ≥ 1-2 hours/week.
