# Supplementary material for: Association between Dietary Patterns and Cardiovascular Risk Factors among Middle-Aged and Elderly Adults in Taiwan: A Population-Based Study from 2003 to 2012
Source: PLoS One. 2016 Jul 1;11(7):e0157745. doi: 10.1371/journal.pone.0157745 (PMC4930186; doi:10.1371/journal.pone.0157745)
Supplement: S2 Table — (PDF) [file pone.0157745.s002.pdf]

Supporting Information

S2 Table. Characteristics of the subjects across quintiles (Q) of vegi-fruit dietary pattern.[1]

	Vegi-fruit dietary pattern					
	**Q1**	**Q2**	**Q3**	**Q4**	**Q5**	***P* for trend**
n	13 803	13 160	13 212	13 215	9575	-
**Dietary pattern score, range**	9 to 14	15 to 16	17 to 18	19 to 21	22 to 42	-
**Sex, % male**	53.8	51.1	52.4	51.5	50.7	< 0.001
**Age, years (SD)**	52.0 (9.9)	51.5 (9.6)	51.7 (9.7)	51.8 (9.8)	52.9 (10.1)	< 0.001
**Education**						< 0.001
**< High school, %**	32.5	27.1	24.7	23.0	23.2	
**High school, %**	34.0	32.8	31.5	30.9	29.5	
**> High school, %**	33.5	40.1	43.8	46.1	47.3	
**Marital status**						< 0.001
**Never married, %**	3.4	2.8	2.6	2.9	2.4	
**Married, %**	84.0	86.3	87.1	87.0	87.3	
**Widows/divorced, %**	12.6	10.9	10.3	10.1	10.3	
**Smoking, %**	28.1	20.3	18.1	15.7	13.6	< 0.001
**Drinking, %**	21.7	18.8	18.1	17.3	16.5	< 0.001
**Physical activity, %**	46.1	59.5	67.5	72.4	79.1	< 0.001
**Cardiovascular disease, %**	4.1	4.2	4.3	4.4	4.6	0.544
**Body mass index, kg/m^2 (SD)**	23.9 (3.3)	23.8 (3.2)	23.8 (3.2)	23.7 (3.1)	23.7 (3.1)	< 0.001
**Waist circumference, cm**	80.1 (10.0)	79.5 (9.6)	79.4 (9.5)	79.0 (9.4)	78.9 (9.4)	< 0.001
**Systolic blood pressure, mm Hg (SD)**	123.9 (19.7)	122.9 (19.4)	122.7 (18.9)	122.7 (18.9)	123.4 (19.4)	0.002
**Diastolic blood pressure, mm Hg (SD)**	74.2 (12.0)	73.9 (11.6)	73.8 (11.6)	73.5 (11.6)	73.8 (11.7)	< 0.001
**Blood lipids**						
**Triacylglycerol, mmol/L (SD)**	1.4 (0.8)	1.3 (0.7)	1.4 (0.7)	1.3 (0.7)	1.3(0.7)	< 0.001
**Total cholesterol, mmol/L (SD)**	5.2 (0.9)	5.2 (0.9)	5.3 (0.9)	5.2 (0.9)	5.1(0.9)	< 0.001
**LDL-C, mmol/L (SD)**	3.1 (0.8)	3.1 (0.9)	3.1 (0.8)	3.1 (0.8)	3.1(0.8)	< 0.001
**HDL-C, mmol/L (SD)**	1.4 (0.4)	1.4 (0.4)	1.4 (0.4)	1.5 (0.4)	1.5 (0.4)	< 0.001
**C-reactive protein, nmol/L (SD)**	25.9 (50.8)	23.0 (44.6)	24.1 (54.8)	22.5 (41.6)	21.8 (38.5)	< 0.001
**Fasting glucose, mmol/L (SD)**	3.9 (1.2)	3.8 (1.2)	3.8 (1.1)	3.8 (1.1)	3.7 (1.1)	< 0.001

[1]Data are expressed as range, %, or mean (SD). General linear regression was used to test for trend with dietary pattern treated as continuous explanatory variable, while χ^2 test was employed for categorical variables across all quintile levels of dietary patterns. Q1: the lowest dietary pattern score of vegi-fruit dietary pattern; Q5: the highest dietary score of vegi-fruit dietary pattern; smoking: ≥ 1-3 times/week; drinking: ≥ 1-2 times/week; physical activity: ≥ 1-2 hours/week.
