# Supplementary material for: Association between Dietary Patterns and Cardiovascular Risk Factors among Middle-Aged and Elderly Adults in Taiwan: A Population-Based Study from 2003 to 2012
Source: PLoS One. 2016 Jul 1;11(7):e0157745. doi: 10.1371/journal.pone.0157745 (PMC4930186; doi:10.1371/journal.pone.0157745)
Supplement: S3 Table — (PDF) [file pone.0157745.s003.pdf]

Supporting Information

S3 Table. Characteristics of the subjects across quintiles (Q) of meat-processed dietary pattern.[1]

	Meat-processed dietary pattern					
	Q1	Q2	Q3	Q4	Q5	*P* for trend
n	13 423	11 631	16 747	11 656	9508	-
**Dietary pattern score, range**	11 to 17	18 to 19	20 to 22	23 to 25	26 to 56	-
**Sex, % male**	39.9	46.7	52.4	59.7	65.4	< 0.001
**Age, years (SD)**	55.2 (10.3)	52.9 (9.9)	51.4 (9.5)	50.3 (9.1)	49.1 (8.8)	< 0.001
**Education**						< 0.001
**< High school, %**	39.4	28.2	23.6	20.1	17.8	
**High school, %**	31.1	32.2	32.7	32.0	31.1	
**> High school, %**	29.5	39.6	43.7	47.8	51.1	
**Marital status**						< 0.001
**Never married, %**	2.4	2.8	3.1	3.0	2.8	
**Married, %**	81.8	85.7	87.1	88.7	88.9	
**Widows/divorced, %**	15.8	11.5	9.8	8.3	8.3	
**Smoking, %**	11.9	15.1	19.2	23.5	31.5	< 0.001
**Drinking, %**	11.9	15.3	18.9	22.6	26.9	< 0.001
**Physical activity, %**	62.8	65.5	64.6	64.6	61.6	< 0.001
**Cardiovascular disease, %**	5.4	4.5	4.1	3.7	3.5	< 0.001
**Body mass index, kg/m^2 (SD)**	23.6 (3.2)	23.6 (3.1)	23.8 (3.2)	23.9 (3.2)	24.2 (3.3)	< 0.001
**Waist circumference, cm**	78.2 (9.4)	78.6 (9.5)	79.3 (9.5)	80.2 (9.6)	81.5 (9.9)	< 0.001
**Systolic blood pressure, mm Hg (SD)**	125.7 (20.7)	123.4 (19.3)	122.5 (19.0)	121.9 (18.4)	121.8 (17.9)	< 0.001
**Diastolic blood pressure, mm Hg (SD)**	70.9 (19.0)	70.5 (18.8)	70.5 (18.9)	70.7 (18.6)	71.2 (18.3)	< 0.001
**Blood lipids**						
**Triacylglycerol, mmol/L (SD)**	1.3 (0.7)	1.3 (0.7)	1.3 (0.7)	1.4 (0.8)	1.4 (0.8)	< 0.001
**Total cholesterol, mmol/L (SD)**	5.2 (0.9)	5.2 (0.9)	5.1 (0.9)	5.2 (0.9)	5.2 (0.9)	< 0.001
**LDL-C, mmol/L (SD)**	3.1 (0.8)	3.1 (0.8)	3.0 (0.8)	3.1 (0.8)	3.2 (0.8)	< 0.001
**HDL-C, mmol/L (SD)**	1.5 (0.4)	1.5 (0.4)	1.5 (0.4)	1.4 (0.4)	1.4 (0.4)	< 0.001
**C-reactive protein, nmol/L (SD)**	24.7 (54.7)	23.4 (47.1)	23.6 (48.8)	22.6 (38.7)	23.4 (40.1)	0.006
**Fasting glucose, mmol/L (SD)**	3.7 (1.1)	3.7 (1.1)	3.8 (1.1)	3.9 (1.2)	4.0 (1.2)	< 0.001

[1]Data are expressed as range, %, or mean (SD). General linear regression was used to test for trend with dietary pattern treated as continuous explanatory variable, while χ^2 test was employed for categorical variables across all quintile levels of dietary patterns. Q1: the lowest dietary pattern score of meat-processed dietary pattern; Q5: the highest dietary score of meat-processed dietary pattern; smoking: ≥ 1-3 times/week; drinking: ≥ 1-2 times/week; physical activity: ≥ 1-2 hours/week.
