# Supplementary material for: Association between Dietary Patterns and Cardiovascular Risk Factors among Middle-Aged and Elderly Adults in Taiwan: A Population-Based Study from 2003 to 2012
Source: PLoS One. 2016 Jul 1;11(7):e0157745. doi: 10.1371/journal.pone.0157745 (PMC4930186; doi:10.1371/journal.pone.0157745)
Supplement: S4 Table — (PDF) [file pone.0157745.s004.pdf]

Supporting Information

S4 Table. Pearson's correlation coefficients (*r*) of cardiovascular disease risk factors.

	Triacylglycerol	Total cholesterol	LDL-C	HDL-C	C-reactive protein	Fasting glucose
**Triacylglycerol**	1.00					
**Total cholesterol**	0.26[1]	1.00				
**LDL-C**	0.11[1]	0.91[1]	1.00			
**HDL-C**	-0.47[1]	0.20[1]	-0.08[1]	1.00		
**C-reactive protein**	0.03[1]	-0.0001	0.02[1]	-0.07[1]	1.00	
**Fasting glucose**	0.62[1]	0.39[1]	0.56[1]	-0.76[1]	0.07[1]	1.00

[1]$p < 0.001$.
