# Supplementary material for: Association between Dietary Patterns and Cardiovascular Risk Factors among Middle-Aged and Elderly Adults in Taiwan: A Population-Based Study from 2003 to 2012
Source: PLoS One. 2016 Jul 1;11(7):e0157745. doi: 10.1371/journal.pone.0157745 (PMC4930186; doi:10.1371/journal.pone.0157745)
Supplement: S5 Table — (PDF) [file pone.0157745.s005.pdf]

Supporting Information

S5 Table. Pearson's correlation coefficients (*r*) between dietary scores, demographic data, lifestyle, or health characteristics and cardiovascular disease risk factors.

	Pearson's correlation coefficient (*r*), *n* = 62,965					
	**Triacylglycerol**	**Total cholesterol**	**LDL-C**	**HDL-C**	**C-reactive protein**	**Fasting glucose**
**Taiwanese dietary scores**	-0.03[3]	-0.07[3]	-0.08[3]	0.02[3]	-0.01[3]	-0.02[3]
**Vegi-fruit dietary scores**	-0.04[3]	-0.04[3]	-0.04[3]	-0.03[3]	-0.03[3]	-0.02[3]
**Meat-processed dietary scores**	0.05[3]	0.02[3]	0.04[3]	-0.08[3]	-0.001	0.04[3]
**Sex**	0.21[3]	-0.01[3]	0.09[3]	-0.39[3]	0.02[3]	0.35[3]
**Age**	0.07[3]	0.11[3]	0.10[3]	-0.006	0.08[3]	0.05[3]
**Education**	-0.03[3]	-0.06[3]	-0.03[3]	-0.06[3]	0.05[3]	0.03[3]
**Marital status**	0.02[3]	0.04[3]	-0.02[3]	0.04[3]	0.02[3]	-0.02[3]
**Smoking**	0.15[3]	-0.01[1]	0.03[3]	-0.20[3]	0.02[3]	0.20[3]
**Drinking**	0.11[3]	0.01	-0.01[2]	-0.05[3]	0.001	0.05[3]
**Physical activity**	-0.03[3]	0.01[2]	0.01[3]	0.02[3]	-0.02[3]	-0.02[3]
**Cardiovascular disease**	0.03[3]	0.003	0.003	-0.02[3]	0.03[3]	0.02[3]
**Body mass index**	0.33[3]	0.11[3]	0.16[3]	-0.35[3]	0.08[3]	0.37[3]
**Waist circumference**	0.39[3]	0.10[3]	0.18[3]	-0.46[3]	0.09[3]	0.46[3]
**Systolic blood pressure**	0.11[3]	0.06[3]	0.05[3]	-0.06[3]	0.03[3]	0.09[3]
**Diastolic blood pressure**	0.14[3]	0.05[3]	0.05[3]	-0.10[3]	0.02[3]	0.12[3]

[1]$p < 0.05$.

[2]$p < 0.01$.

[3]$p < 0.001$.
